# Persistent quality of life impairments in differentiated thyroid cancer patients: results from a monitoring programme

**DOI:** 10.1007/s00259-015-3022-9

**Published:** 2015-03-13

**Authors:** Eva-Maria Gamper, Lisa M. Wintner, Margarida Rodrigues, Sabine Buxbaum, Bernhard Nilica, Susanne Singer, Johannes M Giesinger, Bernhard Holzner, Irene Virgolini

**Affiliations:** 1 Department for Nuclear Medicine, Medical University Innsbruck, Anichstraße 35, 6020 Innsbruck, Austria; 2 Department for Psychiatry and Psychotherapy, Medical University Innsbruck, Innsbruck, Austria; 3 Institute of Medical Biostatistics, Epidemiology, and Informatics, University of Mainz, Mainz, Germany; 4 Netherlands Cancer Institute, Amsterdam, The Netherlands

**Keywords:** Differentiated thyroid cancer, Health-related quality of life, Radioiodine remnant ablation

## Abstract

**Purpose:**

Health-related quality of life (HRQOL) in differentiated thyroid cancer (DTC) research has so far received little attention and available results are conflicting. We studied the HRQOL of radioiodine-naive DTC patients in comparison with the general population (GP), investigated the course of HRQOL up to 30 months after radioiodine remnant ablation (RAA) and sought to identify patient characteristics associated with HRQOL.

**Methods:**

We analysed data from routine HRQOL monitoring at a nuclear medicine department. Between 2005 and 2013, a total of 439 thyroid cancer patients (all histologies) completed the EORTC Quality of Life Questionnaire Core-30 (QLQ-C30) at least once during their treatment at the department. We compared patients’ baseline HRQOL scores before RAA with scores from age-matched and sex-matched controls from the Austrian GP. We then determined the course of HRQOL over the 30 months after RAA and assessed the impact of the following clinical variables on HRQOL: method of thyroid-stimulating hormone (TSH) stimulation, histology (papillary vs. follicular) and disease stage.

**Results:**

A total of 284 patients (mean age 48.3 years, SD 15.0 years; 71.6 % women; 80.7 % papillary type) with a baseline HRQOL assessment before RAA were available. We found clinically meaningful differences in the detriment in patients on almost all domains. These were largest for fatigue (23 points) and role functioning (25 points). Data from 241 patients (mean age 48.6 years, SD 15.9 years; 68.9 % women; 76.3 % papillary type) were included in the longitudinal analysis. Investigating the course of HRQOL, a significant improvement over time was found for role and emotional functioning, fatigue, pain, and dyspnoea. A range of HRQOL scores were improved in patients with exogenous TSH stimulation, but some scores both in patients with exogenous TSH stimulation and in those followed for 30 months, especially fatigue and role functioning, did not reach levels in the GP sample.

**Conclusion:**

Our results show that the favourable prognosis of DTC does not directly translate into good HRQOL in these patients. Persistent restrictions in regaining their normal daily life in terms of work and leisure highlight the importance of more detailed investigation of DTC patients’ wellbeing, support needs, and disease experience.

## Introduction

In the context of differentiated thyroid cancer (DTC) the assessment of patient-reported outcomes, including health-related quality of life (HRQOL), as secondary treatment outcomes has received attention only recently [1, 2]. A low incidence rate of 6.3 per 100,000 per year and a low mortality rate of 0.4 per 100,000 in Europe [3] might have impeded the development of a scientific focus on DTC patients’ HRQOL. Thus, patient-reported information is scarce. Patients in clinical practice often report that they have been told that they have the “good” cancer but that this does not reflect their personal experience with the disease. Studies on long-term HRQOL in DTC patients have shown conflicting results on how long impairment persists beyond the window of active therapy and when patients can expect to reach levels in the general population (GP) again. For example, Schroeder et al. [4] state that patients only experience minor quality of life impairment as long as they do not have to undergo thyroxin withdrawal. Others have found that thyroid cancer survivors still suffer from a range of symptoms and functioning impairments 5 years and longer after diagnosis [5, 6]. Factors influencing HRQOL in DTC patients and survivors have not been sufficiently investigated. There is evidence that exogenous stimulation with recombinant thyroid-stimulating hormone (rhTSH) which facilitates the continued substitution of thyroid hormones is able to prevent hypothyroid-related HRQOL impairment [7–10]. However, for example morbidity and HRQOL impairment as a consequence of life-long TSH suppression therapy have not yet been investigated in detail, but there is literature that suggests that they may be more prevalent than currently assumed [11–13].

During recent years, there has been growing recognition of thyroid cancer patients’ demand for more medical information on their disease and for more psychosocial support [10, 14–17]. Intensified research on HRQOL in this patient group, including the use of cancer-specific HRQOL questionnaires, and more longitudinally designed studies have been called for [18]. The work presented here contributes new knowledge on HRQOL in thyroid cancer patients by analysing longitudinal data from HRQOL monitoring in clinical routine using the widely used cancer-specific HRQOL instrument of the European Organization for Research and Treatment of Cancer (EORTC), the Quality of Life Questionnaire-Core 30 (QLQ-C30) [19]. In contrast to generic HRQOL instruments, which have often been used to investigate thyroid cancer patients’ HRQOL, this questionnaire includes a range of problems experienced by most cancer patients, such as fatigue and pain. In addition, such data collected outside the context of a clinical study is able to provide interesting insights into HRQOL in association with clinical factors, such as the method of TSH stimulation in a routine setting.

Our objective was to explore DTC patients’ HRQOL when admitted for radioiodine remnant ablation (RAA) after thyroidectomy, radioiodine therapy (RAIT) and follow-up, and to identify patient characteristic associated with HRQOL. The two main aims were:

1. To compare DTC patients’ HRQOL profile on the QLQ-C30 at the time of RAA with the profiles of age-matched and sex-matched controls from the GP.
2. To investigate DTC patients’ HRQOL trajectories over a period of 30 months after RAA and to identify patient characteristics associated with HRQOL.

## Materials and methods

### Samples and data collection

We retrospectively analysed data from HRQOL monitoring in DTC patients at the Department of Nuclear Medicine of the Medical University of Innsbruck. According to Austrian law, ethical approval is not required for retrospective analyses of data collected for use in clinical routine.

Patients were treated with ^131^I. Dosages of ^131^I for RAA were selected according to the results of radioiodine uptake and diagnostic scans which were performed 4 weeks after thyroidectomy (usually under endogenous stimulation of TSH). Follow-up examinations, including the radioiodine uptake and diagnostic scan, were performed at 6 and 12 months after thyroidectomy (under endogenous or exogenous stimulation of TSH), and thereafter once per year in patients without further evidence of radioiodine-positive lesions. In patients with radioiodine-positive lesions a follow-up examination including the above-mentioned tests was performed 6 months after each further RAIT.

Patients were invited to complete the QLQ-C30 at each inpatient visit. From 2005 until 2011 HRQOL data were collected on a paper and pencil basis and from 2011 with a computer-based system using the software CHES (Computer-based Health Evaluation System) [20]. Sociodemographic and clinical data were either directly entered into the CHES system in case of electronic assessment or collected from medical records for questionnaires from the period of paper and pencil assessment.

General criteria for data to be included in the present analysis were:

- Histologically confirmed and ^131^I-sensitive DTC
- date of diagnosis not before 1 January 2005 (due to availability of electronic patient data)

Additional inclusion criterion for aim 1 was:

- Admission for RAA after thyroidectomy

Additional inclusion criteria for aim 2 were:

- Minimum two assessments per patient
- Assessments up to 30 months from diagnosis

The GP sample was drawn in 2002 by randomly selecting 2,000 persons from the official telephone directory. The response rate was 50.6 %. Representativeness was checked by comparing the distribution of sociodemographic variables in the sample with data from Statistics Austria. The same sample was used for collecting reference data for the Functional Assessment of Cancer Therapy – General (FACT-G) and has been described in detail in the respective publication [21]. Individuals from the GP sample (without cancer) were age-matched and sex-matched to the DTC sample.

### HRQOL questionnaire

HRQOL was assessed using the EORTC QLQ-C30 [19] as not only is it the most widely used cancer-specific HRQOL instrument in Europe, but was also expected to capture issues relevant to thyroid cancer patients. It comprises 30 questions building five functioning scales (physical, social, role, emotional, cognitive), nine symptom scales (fatigue, nausea/vomiting, pain, dyspnoea, sleep disturbances, appetite loss, constipation, diarrhoea, and financial impact), and a scale for global quality of life (QOL). Scoring was done according to the QLQ-C30 manual. Raw scores were transformed to a scale of 0 – 100 with 100 reflecting the best score possible for functioning scales and the worst score for symptom scales.

### Statistical analysis

#### Patient characteristics and sampling bias

Patient characteristics are provided as mean and median percentages with standard deviations (SD) and interquartile ranges (IQR). As we selected subsamples from a larger dataset of thyroid cancer patients, the degree of selection bias was assessed by comparing the patient characteristics with data from the Tyrolean Tumour Registry [22]and comparing included and excluded patients in terms of age, sex and clinical characteristics.

#### Analysis for aim 1

Analysis of variance was used to evaluate differences in HRQOL between the DTC patients and the GP sample. Variables included were group (DTC vs. GP), sex and age. To assess if the effects of age and sex were disease-specific or similar to age and sex differences in the GP, interactions between group and age as well as group and sex were investigated.

#### Analysis for aim 2

HRQOL trajectories and patient characteristics associated with HRQOL were investigated using mixed linear models due to their ability to handle unbalanced data. This was necessary as we did not have HRQOL assessments for all patients at all time points The domains of the QLQ-C30 were included as dependent variables. Age, sex and clinical variables (histology, stage, method of TSH stimulation and time since RAA) were included as independent variables and first tested in univariate analysis. The number of cycles of RAIT and the cumulative dose of ^131^I were not included as they were time-dependent and confounded by the variable time since RAA. Characteristics with a significant association with HRQOL scores in univariate analyses were included in multivariate models and excluded stepwise if they were no longer significant. Interaction effects were investigated for sex/age and clinical variables. In addition, we accounted for correlations between assessments and included individual intercepts as random factors.

#### Meaningful changes

For the assessment of the clinical meaningfulness of HRQOL differences, we referred to the standard guidelines of Osoba et al. [23]. According to these guidelines, a change of 5 – 10 % in the scale range can be perceived as a meaningful change by a patient. For the scales of the QLQ-C30, this results in defining a difference of 5 – 10 points as a “small” difference, 10 – 20 points as a “moderate” difference and >20 points as a “large” difference.

All statistical analyses were done using SPSS 21.0.

## Results

### Patient characteristics

**Fig. 1:**
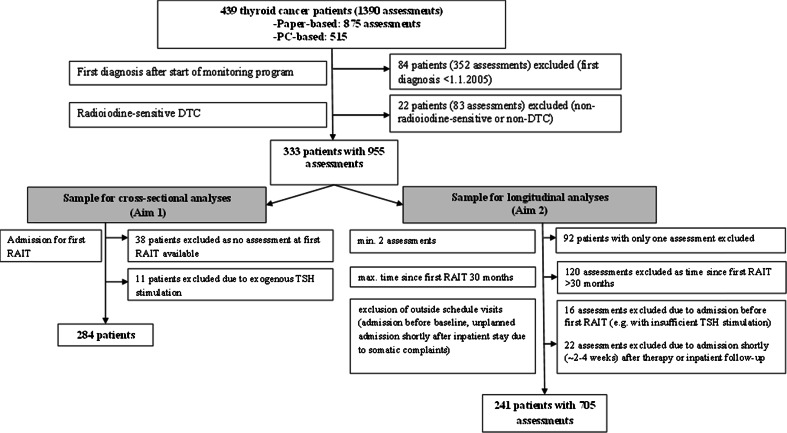
Flow diagram of the patient selection process

The flow diagram in Fig. 1 shows the patient selection process. Between January 2005 and September 2013, 439 thyroid cancer (DTC and non-DTC) patients completed the HRQOL questionnaire at least once. Altogether, these patients completed 1,390 assessments. Applying the criteria described in the Statistical analysis section, 295 radioiodine-naive patients were eligible for cross-sectional analysis in aim 1. At our centre, patients usually undergo endogenous TSH stimulation for RAA. Thus, only 11 of these 295 patients had exogenous TSH stimulation for RAA. As this number was too small to be included as a factor in the analyses, these 11 patients were excluded, leaving 284 for comparison with the GP sample. Their mean age was 48.3 years (SD 15.0 years) and 71.6 % were women. Controls from the GP sample were age-matched and sex-matched to the patient sample.

For the longitudinal analysis, 241 patients were eligible, i.e. had completed the questionnaire at least twice. The mean age of the patients was 48.6 years (SD 15.9 years), and 68.9 % were women. The patient characteristics are shown in Table 1, and the details of the 705 assessments performed in the 241 patients included in the longitudinal analysis are shown in Table 2.

**Table 1:** Patient characteristics

Characteristic	Aim 1: cross-sectional comparison	Aim 2: longitudinal analyses of HRQOL		
	RAIT-naive patients (*N* = 285)	Included patients (*N* = 241)	Excluded patients^a^ (*N* = 92)	*P* value^b^
Age (years), mean ± SD	48.3 ± 15.0	48.6 ± 15.9	47.8 ± 13.0	0.563
Sex: female, *n* (%)	204 (71.6)	166 (68.9)	63 (68.4)	0.559
Histology, *n* (%)				
Papillary	230 (80.7)	184 (76.3)	83 (91.2)	0.002
Follicular	55 (19.3)	57 (23.7)	9 (9.8)	
Tumour stage, *n* (%)				
I + II	217 (76.1)	181 (75.1)	72 (78.3)	0.548
III + IVa	60 (21.1)	53 (22.0)	18 (19.6)	
Missing	8 (2.8)	7 (2.9)	2 (2.2)	
Assessment method, *n* (%)				
Paper and pencil	139 (48.8)			
Computer-based	146 (51.2)			

^a^The reasons for inclusion/exclusion are shown in the Statistical analysis section and the flow diagram in Fig. 1

^b^ *p* value is from the *t*-test or *χ* ^2^ − test for group comparisons

**Table 2:** Details of 705 assessments performed in the 241 patients included in the longitudinal analysis

Assessments	Value
Time since diagnosis (months), mean ± SD	13.3 ± 11.6
Number of RAIT cycles, median (IQR)^a^	1.0 (1.0)
Number of assessments at each time-point, *n*	
Baseline	198
6 months	195
12 months	104
18 months	94
24 months	53
30 months	61
Admission reason, *n* (%)	
Therapy	261 (37.0)
Follow-up	444 (63.0)
Method of TSH stimulation, *n* (%)	
Exogenous	46 (6.5)^b^
Endogenous	657 (93.2)
Missing	2 (0.3)
Assessment method, *n* (%)	
Paper and pencil	424 (60.1)
Computer-based	281 (39.9)

^a^Therapeutic doses 3.7 and 7.4 GBq

^b^41 patients with 46 assessments

### Assessment of sampling bias

The subsamples drawn for analysis appeared to be comparable to the patients from the Tyrolean Tumour Registry concerning sex (74 % vs. 72 % and 69 % in our sample) and age (50 years vs. 48 years in our samples). In addition, the numbers of patients in our subsamples diagnosed with localized disease (76 % and 75 %) were comparable to numbers from the Tyrolean Tumour Registry (75 %). In our sample there were more patients with distant disease (21 % and 22 %) than among the patients from the Tyrolean Tumour Registry (11 %). Information on histology could not be obtained from the registry, but percentages for papillary type and follicular type in our sample largely reflected the usual incidence patterns for thyroid cancer (76 % papillary, 18 % follicular) [24]. Patients included in the longitudinal analysis were not different from the excluded patients in terms of age (*p* = 0.563), sex (*p* = 0.559) and stage (*p* = 0.548). The samples differed in terms of histology (*p* = 0.002). For details see Table 1.

### Aim 1: Comparison of DTC patients’ HRQOL with the GP

Comparisons of DTC patients before RAA with Austrian GP scores showed that the patients’ HRQOL was significantly worse (*p* < 0.001 and *p* = 0.014) on all domains except cognitive functioning, diarrhoea and financial impact. Differences that were statistically significant were also clinically meaningful (≥5 points difference) except pain and nausea/vomiting. Large differences were found for role functioning (25 points) and fatigue (23 points). Moderate differences were found for sleep disturbances (15 points), global QOL (15 points) and dyspnoea (11 points). Differences for physical, social and emotional functioning, appetite loss and constipation were small (7 – 10 points).

**Fig. 2:**
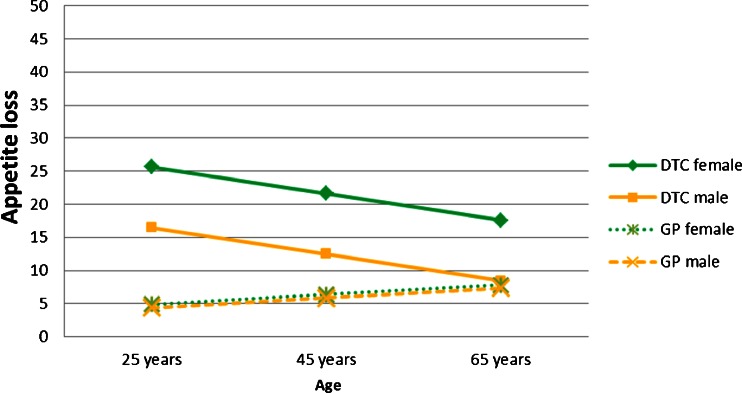
Interaction between patient samples (GP vs. DTC patients) and sex and age for appetite loss

To test if the influence of sex and age on HRQOL were different in patients and the GP, interaction effects were also considered in the comparison of the two groups. Significant interactions between group and sex were found for role, social and cognitive functioning as well as appetite loss. These showed that on these domains female patients had significantly more problems than male patients, while the difference between men and women in the GP was only marginal (as an example, see the interaction effect for appetite loss in Fig. 2). Significant interactions between group and age were found for social functioning, pain and appetite loss. The effects indicated that social functioning was decreased in patients but did not additionally decrease with age in the patient group in contrast to the GP. For pain the curve of decline with age was flatter in patients than in the GP and appetite loss became less severe with age in patients, whereas it did not show a relevant change in the GP (as an example, see the interaction effect for appetite loss in Fig. 2).

Table 3 shows the marginal means for DTC patients and the GP including impact of age and sex, and parameters for interaction effects for all domains.

**Table 3:** Sex-adjusted and age-adjusted marginal means for patients and the GP with 95 % confidence intervals and parameters for interaction effects

Domain	Marginal means (95 % CI)				Interactions effects			
	Patients	General population	Mean difference^a^	*p* value	Sample × sex^b^	*p* value	Sample × age^c^	*p* value
Physical functioning^d^	82 (81 – 84)	90 (88 – 92)	**8**	<0.001				
Role functioning	62 (59 – 66)	87 (83 – 90)	**25**	<0.001	−11	0.033		
Social functioning	73 (70 – 77)	84 (81 – 87)	**9**	0.001	−11	0.026	0.33	0.026
Emotional functioning^e^	63 (60 – 66)	72 (69 – 75)	**9**	<0.001				
Cognitive functioning^e,d^	80 (77 – 83)	83 (80 – 86)	3	0.138	−10	0.017		
Global QOL^d^	61 (58 – 64)	76 (74 – 79)	**15**	<0.001				
Fatigue^e^	45 (2 – 47)	22 (19 – 25)	**23**	<0.001				
Nausea/vomiting	7 (5 – 9)	4 (3 – 6)	3	0.013				
Pain^d^	22 (18 – 25)	20 (17 – 23)	2	0.014			−0.33	0.017
Dyspnoea^d^	22 (20 – 25)	9 (7 – 12)	**11**	<0.001				
Sleep disturbance^d^	39 (36 – 43)	24 (21 – 27)	**15**	<0.001				
Appetite loss^e^	16 (13 – 19)	6 (4 – 9)	**10**	<0.001	8.7	0.040	−0.28	0.033
Constipation^e^	15 (12 – 18)	8 (5 – 11)	**7**	0.001				
Diarrhoea^e^	8 (5 – 10)	9 (7 – 11)	1	0.532				
Financial impact^d^	12 (9 – 15)	10 (7 – 12)	2	0.335				

^a^Clinically meaningful differences in bold type

^b^Reference group: male

^**c**^Reference group: patient

^d^Main effect of age worse scores with increasing age (*p* ≤ 0.030):

^e^Significant main effect of sex worse scores in women (*p* ≤ 0.007):

### Aim 2: Course of HRQOL in DTC patients

**Fig. 3:**
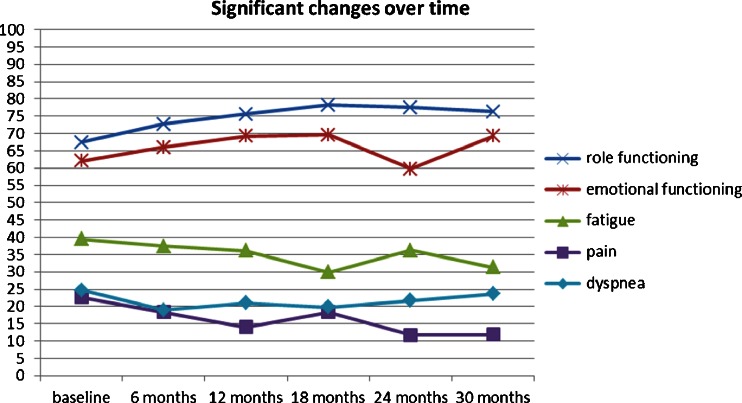
Scales with significant changes over time (adjusted for effects of age, sex and method of TSH stimulation)

**Fig. 4:**
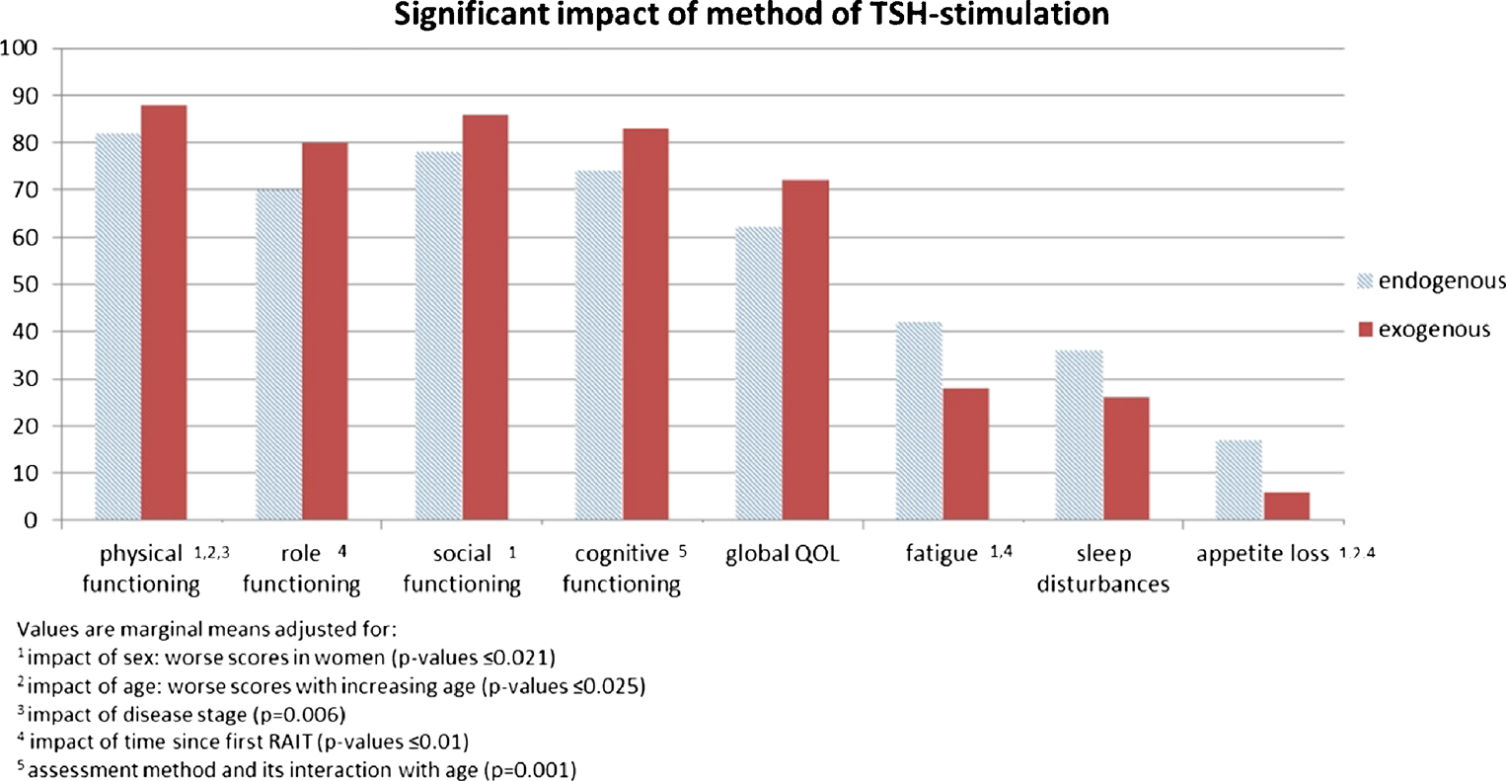
Scales with a significant impact of method of TSH stimulation

Variables with a significant association with HRQOL in multivariate models were time since RAA, disease stage and the method of TSH stimulation (*p* values between 0.006 and 0.014). A change over time (over 30 months after RAA) was found for role and emotional functioning, fatigue, pain and dyspnoea. Despite variations between time-points (mostly below or barely reaching the 5-point cut-off except for emotional functioning) the changes were largely linear improvements. The largest improvement between baseline and follow-up at 30 months was found for pain (11 points). Role functioning improved 9 points, emotional function 7 points, and fatigue 8 points (see Fig. 3). Localized disease was associated with better physical functioning only (7 point difference compared with patients with distant disease). Exogenous TSH stimulation was associated with better physical functioning (6 points), role functioning (10 points), cognitive functioning (8 points) and global QOL (10 points), and less fatigue (14 points), sleep disturbance (10 points) and appetite loss (11 points; Fig. 4).

While pain scores even fell below the level in the GP over 30 months after RAA, role functioning, fatigue, and dyspnoea scores remained clearly below the level in the GP (defined as a maximum difference of 5 points) during the entire assessment period, also under exogenous TSH stimulation.

Table 4 shows sex-adjusted and age-adjusted marginal means for clinical subgroups.

**Table 4:** Marginal means for clinical subgroups with significant score differences

Domain	Marginal means (95 % CI)			
	Method of TSH stimulation		Stage	
	Endogenous	Exogenous	Localized	Distant
Physical functioning^a,b^	82 (78 – 85)	88 (83 – 92)	88 (82 – 92)	81 (79 – 85)
Role functioning^c^	70 (66 – 73)	80 (71 – 88)		
Social functioning^a^	78 (75 – 81)	86 (79 – 93)		
Emotional functioning^a,c^				
Cognitive functioning^d^	74 (71 – 77)	83 (76 – 90)		
Global QOL	62 (60 – 65)	72 (65 – 78)		
Fatigue^a,c^	42 (20 – 35)	28 (39 – 46)		
Nausea/vomiting				
Pain^c^				
Dyspnoea				
Sleep disturbance^a,b,c^	36 (32 – 40)	26 (17 – 35)		
Appetite loss	17 (14 – 19)	6 (0 – 14)		
Constipation^a^				
Diarrhoea				
Financial impact				

^a^Adjusted for impact of sex: worse scores in women (*p* ≤ 0.021)

^b^Adjusted for impact of age: worse scores with increasing age (*p* ≤ 0.025)

^c^Adjusted for time since diagnosis (*p* ≤ 0.01)

^d^Adjusted for assessment method and its interaction with age (*p* = 0.001)

## Discussion

In the present investigation we analysed the HRQOL of DTC patients after thyroidectomy undergoing RAA and RAIT and during follow-up using data from HRQOL monitoring in clinical routine. Such data have so far been scarce and provide valuable additional information to findings from clinical studies.

In a cross-sectional study we compared HRQOL scores in DTC patients at the time of their RAA with scores in the Austrian GP. In addition, we analysed longitudinal data up to 30 months after RAA and investigated the effects of histology (papillary vs. follicular), disease stage, method of TSH stimulation and time since RAA on HRQOL scores. The cumulative dose of ^131^I has shown to be a clinical predictor of HRQOL [25] but could not be included into our multivariate models as it was highly confounded by time from baseline. Thus, the effects of time since RAA could very well be attributable to this clinical variable, but statistically could not be assigned to either of them due to collinearity. In all analyses we adjusted for the effects of age and sex, i.e. the effects we found cannot be explained by the facts that women usually report worse HRQOL and that the majority of DTC patients are women.

In the cross-sectional comparison of HRQOL scores in DTC patients and the scores in the GP, patients reported worse HRQOL on almost all domains of the QLQ-C30. In particular, large differences were found for role functioning (25 points; comprising questions on the ability to perform usual work and leisure activities), fatigue (23 points), sleep disturbance (15 points), and global QOL (15 points; comprising questions on subjective global health state and QOL). Against our expectations from clinical experience, there was no overall impairment of cognitive functioning in patients. However, there was a significant difference in cognitive functioning between DTC patients and the GP, but in women only. A similar interaction effect was found for role functioning and social functioning (comprising questions on ability to participate in social activities and fulfil social roles) and appetite loss.

These results were not surprising insofar as all of our DTC patients in this treatment phase were in a hypothyroid state. It is well known that hypothyroidism causes a broad range of physical as well as psychological symptoms, such as constipation, weight gain, fatigue, depression and slowed cognition, and that associated distress is high [26, 27]. However, the magnitude of the effect was still striking as it is comparable to that in other cancer diagnoses which are usually considered much more impairing, such as laryngeal cancer [28]. Furthermore, our longitudinal analyses indicated that some issues were influenced by a hypothyroid state to the extent we had expected. Emotional functioning (comprising feelings of worry, anxiousness, nervousness and irritability) in our sample had improved to the level in the GP (defined as a difference <5 points) after 12 months, had worsened at 24 months, and had recovered again at 30 months, but without significant effect of the method of TSH stimulation. Similar fluctuations over time were observed for fatigue scores, which were significantly lower under exogenous TSH stimulation but without reaching the level in the GP level during the observation period. Likewise, role functioning did not reach the level in the GP level either over time or under exogenous TSH stimulation. However, in line with previous reports that exogenous TSH stimulation is associated with better HRQOL especially with regard to functioning (physical, social, role and emotional), sleep disturbance and fatigue [8, 29], our patients with endogenous TSH stimulation were also at risk of low HRQOL (on the domains physical, role, social and cognitive functioning, global QOL, fatigue, sleep disturbance and appetite loss). Yet the conclusion that HRQOL impairment is minor in DTC patients as long as they do not have to undergo hormone withdrawal [4] cannot be supported by the present data.

There are conflicting reports in the literature as to when a restored HRQOL can be expected. For example, Taieb et al. found that HROQL is restored shortly after surgery [30] and Crevenna et al. found significant improvements at 1 year after diagnosis and beyond [5]. Our results, on the one hand, showed significant improvements after 12 months for some domains. On the other hand, they also support reports of prolonged posttreatment fatigue in thyroid cancer patients and survivors [31]. Physiological explanations discussed are related to TSH suppression during posttreatment care. Fatigue might be a result of subclinical hyperthyroidism [32] which again possibly causes a permanent change in the autonomic nervous system [33]. It has also been suggested that the optimal preoperative TSH level might be different from the target value of TSH suppressive therapy [34]. Husson et al. found that fatigue in short-term and long-term survivors of thyroid cancer is strongly associated with thyroid-specific HRQOL, including sympathetic problems and emotional distress [35]. Our own data showed similar courses of fatigue and emotional distress over time. These results suggest that, aside from identifying physiological causes, the emotional aspect of fatigue might require more attention in the management of thyroid cancer patients as psychological coping mechanisms may play an important role here.

Our results on the comparison of patient scores and scores from the GP are largely in line with the results from a previous study on HRQOL in thyroid cancer patients during inpatient rehabilitation conducted in Germany [15]. The German study clearly showed larger differences between patients and the GP on QLQ-C30 domains. The scores differed from our data by >5 points, except for global QOL, nausea/vomiting, appetite loss, constipation and diarrhoea. In particular, cognitive and emotional problems, pain and financial difficulties were more pronounced in the German patient group (the scores differed from our data by ≥19 points). The fact that patients with higher disease burden are more likely to make use of inpatient rehabilitation could be one explanation for these large differences. In addition, rehabilitation prolongs absenteeism from work which might cause additional financial strain. It is also not unusual that psychosocial distress in particular is aggravated directly after completion of active therapy.

The impact of sex and age found in our sample is in line with previous reports [5, 15, 25, 36], as we also found more impairment in women than in men and decreasing scores with increasing age on a range of domains. Most of these effects were not disease-specific, but rather reflected sex and age differences that can also be observed in the GP. This is an important issue when interpreting such effects in patient samples [37]. In addition, disease-specific patterns of the impact of age were found for social functioning, pain and appetite loss. While scores in patients were significantly worse, their decline with age was less pronounced than in the GP.

Despite that the fact that our results were inherently consistent and bias assessment did not cause severe concern, the present analysis had some limitations that restrict the generalization of the findings. Firstly, in our samples the number of patients with follicular cancer type was about 10 % higher than in the GP. We did not, however, find an effect of histology in multivariate analysis, indicating that other factors were more strongly associated with HRQOL. Secondly, the number of patients in the monitoring programme decreased with time, and thus the results may have been biased by the selection of patients. However, there was no significant difference in stage of disease between earlier and later assessment time-points, and the majority of patients at the 30-month follow-up had received one or two cycles of RAIT. Thus, there is no indication that patients with more severe disease participated longer in HRQOL monitoring. Thirdly, there was an unbalanced distribution of assessments with exogenous and endogenous TSH stimulation which limited the validity of the results of comparisons between the two methods. Therefore, analyses including this factor were rerun but resulted in only minor changes in the remaining model parameters (results not shown).

Fourthly, the distribution of paper and pencil assessments and of electronic assessments suggest more missing data during the period of paper and pencil assessment. Therefore, we investigated the influence of the assessment method on HRQOL scores and found that older patients scored worse on cognitive functioning when assessed using the computer-based system (8 points difference). This finding may indicate some selection bias. A plausible explanation seems to be that the inclusion of patients in the HRQOL monitoring programme might have been affected by subjective factors. Clinical personnel who, since electronic assessment can immediately access a patient’s HRQOL scores and the information can be used in the communication with the patient, might have become increasingly motivated to include as many patients as possible. In addition, presentation of questions on a tablet computer might be advantageous for cognitively impaired patients as there are usually only two or three questions per page in a large font. Detailed analysis on the impact of assessment method on cognitive functioning in the entire monitoring sample is warranted.

It has also to be kept in mind that our Austrian reference data were collected in 2002 and values may have changed in the meantime. Also we cannot provide information on possible differences between Tyrolean and Austrian GP values, which if they do exist, would results in either overestimation or underestimation of thyroid cancer patients’ HRQOL impairment. Our reference values were, however, consistent with the EORTC QLQ reference values for the GP published in 2008 [38] and, thus, seem to be sufficiently sensitive. In addition, in a recent update of the German reference values for the QLQ-C30 from the year 2001 changes were small and not clinically relevant for most scales, except for fatigue, sleep disturbances and pain. These symptoms were more severe in the new GP sample, which according to the authors, may be attributed to a sampling bias [39].

In conclusion, our results provide further evidence that DTC patients’ burden from symptoms and functioning impairments is unrelated to the favourable clinical outcome. Nonetheless, thyroid cancer is still often labelled as the “good” cancer with only minor impairments so that patients may feel that their concerns are being trivialized and that they lack information and support [16, 40]. Psychosocial distress as well as persistent problems with fatigue and possibly resulting difficulties at work and during leisure time are frequently overlooked in clinical practice and often falsely attributed to hypothyroidism only. Thyroid cancer patients with HRQOL impairments which persist quite some time after treatment are not uncommon. This is important information for treating physicians, especially as the “average” thyroid cancer patient is usually seen by representatives of a range of different medical professions and there is rarely continuity of care during the treatment and follow-up process. Acknowledging thyroid cancer patients’ burden is essential in improving their care, especially when it comes to psychosocial issues. Finally, our results highlight the need for more systematic investigations of DTC patients HRQOL in order to provide patients with reliable information about what they need to expect when living with a DTC diagnosis and the sequelae of treatment.
